# Effect of the Number of Removed Lymph Nodes on Survival in Patients with FIGO Stage IB-IIA Cervical Squamous Cell Carcinoma following Open Radical Hysterectomy with Pelvic Lymphadenectomy: A Retrospective Cohort Study

**DOI:** 10.1155/2021/6201634

**Published:** 2021-04-14

**Authors:** Qinhao Guo, Yong Wu, Hao Wen, Xingzhu Ju, Xiaohua Wu

**Affiliations:** ^1^Department of Oncology, Shanghai Medical College, Fudan University, 130 Dong-An Road, Shanghai 200032, China; ^2^Department of Gynecologic Oncology, Fudan University Shanghai Cancer Center, 270 Dong-An Road, Shanghai 200032, China

## Abstract

**Objective:**

To determine whether the number of removed lymph nodes (RLN) is associated with survival in patients with International Federation of Gynecology and Obstetrics (FIGO) stage IB-IIA cervical squamous cell carcinoma (CSCC).

**Methods:**

We reviewed the medical records of FIGO stage IB-IIA CSCC patients who underwent standardized radical hysterectomy with pelvic lymphadenectomy (RHPL) in our center between 2006 and 2014. The X-tile software was performed to calculate the optimal grouping of cutoff points for RLN. The impact of RLN on progression-free survival (PFS) and overall survival (OS) was analyzed using Cox regression analysis.

**Results:**

Among 3,127 patients, the mean number of RLN was 22, and positive lymph node (LN) was found in 668 (21.4%) patients. X-tile plots identified “21” and “16” as the optimal cutoff value of RLN to divide the patients into two groups in terms of PFS and OS separately. In all patients, the number of RLN was not associated with PFS (*P*=0.182) or OS (*P*=0.193). Moreover, in both LN positive and negative patients, the number of RLN was not associated with either PFS (*P*=0.212 and *P*=0.540, respectively) or OS (*P*=0.173 and *P*=0.497, respectively). Cox regression analysis showed that the number of RLN was not an independent prognostic factor for PFS or OS.

**Conclusion:**

If standardized RHPL was performed, the number of RLN was not an independent prognostic factor for survival of patients with FIGO stage IB-IIA CSCC.

## 1. Introduction

Despite efficient screening and vaccination [1, 2], cervical cancer continues to be the fourth most prevalent cause of cancer-related death in women worldwide, especially in developing countries [3]. Radical hysterectomy with pelvic lymphadenectomy (RHPL) is the standard surgical treatment for early-stage cervical cancer, in particular, International Federation of Gynecology and Obstetrics (FIGO) 2009 Stage IB-IIA disease [4]. Cervical squamous cell carcinoma (CSCC), the most common pathological type of cervical cancer, accounts for about 80–90% but is less likely to develop lymph node metastasis than adenocarcinoma and adenosquamous cell carcinoma [5].

Most tumors typically spread multidirectionally via the lymphatic system; hence, lymph nodal (LN) status is a strong prognostic factor for survival in patients with cervical cancer [6]. The goal of standardized lymphadenectomy is to provide an accurate pathologic diagnosis of LN status and a possible therapeutic advantage [7]. Given the long natural history of LN dissections for tumors, it was our instinct that a more thorough lymphadenectomy should increase the number of removed lymph nodes (RLN), and the more nodes retrieved, the more likelihood of the better survival. For non-small cell lung cancer [8] or bladder cancer [9], previous studies had reported the survival benefits for patients who had undergone the removal of an increased number of LN. These authors believe that a more extensive lymphadenectomy could improve the accuracy of pathologic diagnosis by improving the detection of lymph node status, and this was supposed to have possible therapeutic benefits for patients. However, extensive lymphadenectomy could increase the surgical trauma, which was the leading cause of postoperative complications [7, 10], and some recent studies demonstrated a more extensive lymphadenectomy does not improve survival after surgery for esophageal cancer [11], prostate cancer [12], and endometrial cancer [13], which even challenged the current clinical guidelines.

For cervical cancer, studies on the possible benefit of a more extensive lymphadenectomy were limited. Some reports indicated the possible survival benefits from removing more LN among cervical cancer patients [14]. However, recent studies demonstrated conflicting results on the same issue [15, 16]. Furthermore, the majority of low-risk early-stage cervical cancers (FIGO stages IA to IIA) do not present with LN metastases, and the prevalence of pelvic node metastases for stage I and stage II cervical cancer patients was 0–25.3% and 24–31%, respectively [16]. But the current situation is that almost all LN negative patients also underwent systematic lymphadenectomy, which might destroy the equilibrium of the immune system. Regional lymph nodes usually raise a tumor-directed immune response in defence against tumor invasion at the early stage [17]. Could these patients benefit from complete pelvic lymphadenectomy? Thus, the possible therapeutic value of a more extensive lymphadenectomy for the treatment of early-stage cervical cancer remains an open question.

To date, there is no relevant report to describe the possible benefit of a more extensive lymphadenectomy for patients with early-stage CSCC. Through the survival analysis of our patient's cohort treated with standard RHPL, we investigated whether the number of RLN is an independent prognostic factor for survival of patients with FIGO stage IB-IIA CSCC.

## 2. Materials and Methods

### 2.1. Patients

The data of patients with IB or IIA CSCC with FIGO (2009) stage who underwent abdominal radical hysterectomy ± bilateral salpingo-oophorectomy and pelvic lymphadenectomy between 2006 and 2014 were reviewed retrospectively. By checking the surgical records, all surgeries were performed by at least two experienced gynecological oncologists in our center by using the standardized RHPL. All LN and fatty tissues were supposed to be removed between the external and internal iliac arteries, from the bifurcation of the common iliac artery up to the circumflex vein and above the obturator nerve. In our center, para-aortic lymph node resection was performed if intraoperative palpation suggested para-aortic lymph node involvement or if intraoperative frozen section examination showed positive common iliac LN. In order to avoid the influence of the number of RLN, we excluded the patients with para-aortic lymphadenectomy. Also, patients who were with comorbidities and previous history of cancer and received neoadjuvant chemotherapy or preoperative radiotherapy, died within 30 days after surgery, and had a follow-up time less than three months were excluded from this study. The criteria for adjuvant treatment after surgery were ≥2 intermediate-risk factors (tumor diameter ≥4 cm, ≥1/2 depth of stromal invasion, and lymph-vascular space invasion (LVSI)) or ≥1 high-risk factor (positive parametrium, positive LN, and involved surgical margins). Patients with only one intermediate-risk factor were exempted from any adjuvant treatment. After treatment, patients were followed up every three months for the first two years, every six months for the next three years, and once per year after that. If adjuvant therapy was administrated, a monthly follow-up was ensured for the first six months after surgery. Follow-up visits included pelvic examinations, abdominal ultrasonography, chest X-ray, routine blood test, serum squamous cell carcinoma antigen (SCC-Ag), vaginal cytology, computed tomography (CT), or magnetic resonance imaging (MRI) scan. Written informed consent was obtained from all the participants preoperatively. The study was approved by the ethics committee at our center.

Demographic and clinical characteristics of the patients, along with survival data, were assessed. The pathologic evaluation included patients' age, menopausal status, FIGO stage, tumor diameter, depth of stromal invasion, LVSI, parametrial involvement, vaginal margin invasion, LN metastasis, and number of RLN. The gynecology-dedicated pathologists dissected fat and lymphatic specimens to identify lymph nodes, and the total number of RLN in each case was obtained from the descriptions of pathologists. Separate analyses were performed for LN positive and LN negative patients.

### 2.2. Statistical Analyses

The RLN cutoff points were determined using the X-tile program [18], which identified the cutoff value with the minimum *P* values from log-rank *χ*2 statistics for the categorical RLN in terms of progression-free survival (PFS) and overall survival (OS). PFS was defined as the time of primary surgery to the first disease progression and OS was defined as the interval from the date of primary surgery to death or the latest observation. Pearson's *χ*2 test was used to analyze the association between the number of RLN and clinical and pathological features in CSCC patients. Kaplan-Meier survival curves were used to compare the PFS or OS between different groups of patients. Hazard ratios (HR) with 95% confidence intervals (CI) were calculated by univariate and multivariate analyses using Cox proportional hazards models to evaluate the prognostic factors for survival. To test whether the chosen cutoff values impacted the relative performance of RLN, we analyzed the number of RLN as both continuous and categorical variables. *P* < 0.05 was considered to indicate statistical significance. All statistical analyses were performed with SPSS (Version 22.0; SPSS Inc, Chicago, Ill).

## 3. Results

### 3.1. Patient Demographics

**Figure 1:**
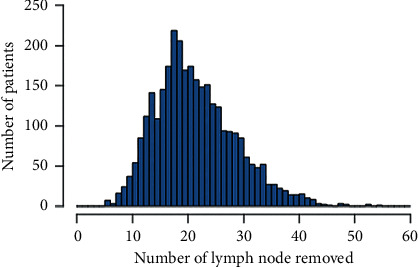
Distribution of lymph nodes removed.

A total of 3,127 patients who met the inclusion criteria were enrolled in the analysis. The median age of these patients was 47 (range: 23–97), and 32.8% (1,025 of 3,127) of patients were in menopause. Among the patients, 668 (21.4%) cases had LN metastasis, and parametrial area and vagina were involved in 4.6% and 3.6% of cases, respectively. The mean follow-up time was 40.1 months (range: 4–116 months). All the patients underwent radical abdominal surgery, of whom 100% of the patients received the pelvic lymphadenectomy. There were 1,984 patients (63.4%) receiving postoperative adjuvant therapy, including adjuvant radiotherapy and chemotherapy. The distribution of the numbers of RLN in the cohort was shown in Figure 1. The total numbers of dissected lymph nodes were up to 68,326, and the mean number of RLN was 22 (range: 6–55).

### 3.2. The Optimal Cutoff Value for RLN Count Calculated by X-Tile

**Figure 2:**
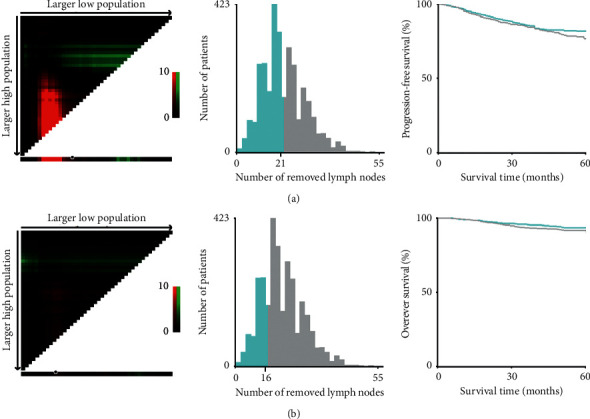
X-tile analysis of PFS (a) and OS (b) according to the number of RLN. PFS, progression-free survival; OS, overall survival; RLN, removed lymph nodes.

Patients' information of PFS and OS were input to X-tile software with the number of RLN, respectively, and the optimal cutoff points for the RLN were analyzed by the computer program. The cutoff point of RLN for PFS was “21”, which divided the patients into two groups: “RLN ≤21” group and “RLN >21” group. The RLN for OS was divided into two groups with “16” as the boundary: “RLN ≤16” and “RLN >16” group (Figure 2).

### 3.3. Clinical and Pathological Characteristics and Survival Analysis

**Figure 3:**
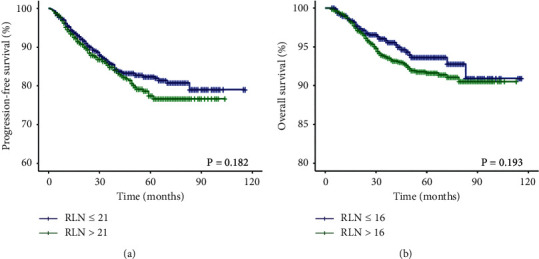
Kaplan-Meier plot of PFS (a) and OS (b) according to the number of RLN in all the patients. PFS, progression-free survival; OS, overall survival; RLN, removed lymph nodes.

Table 1 provides a complete display of the clinical and demographic characteristics of the cohort, stratified by number of RLN. For PFS, 1,675 (53.6%) cases had 6–21 nodes removed, and 1,452 (46.4%) cases had 22–55 nodes removed, and for OS, 733 (23.4%) cases had 6–16 nodes removed, and 2,394 (76.6%) cases had 17–55 nodes removed. When the groups were compared separately, there was a significant difference in tumor diameter (*P*_PFS_ < 0.001 and *P*_OS_ < 0.001), but no significant differences were found in patients' age, menopausal status, FIGO stage, depth of stromal invasion, LVSI, parametrial involvement, or vaginal margin invasion. The Kaplan-Meier survival curve showed that RLN was not associated with PFS (*P*=0.182, Figure 3(a)) or OS (*P*=0.193, Figure 3(b)), respectively.

### 3.4. Cox Proportional Hazards Regression Analysis

As shown in Table 2, we used Cox proportional hazards regression model to assess the relationship between clinical and pathologic factors with PFS and OS in the entire cohort; the univariable Cox model results showed that patients' age (*P*=0.014), menopausal status (*P*=0.004), FIGO stage (*P* < 0.001), tumor diameter (*P* < 0.001), depth of stromal invasion (*P* < 0.001), LVSI (*P* < 0.001), parametrial invasion (*P* < 0.001), vaginal margin invasion (*P* < 0.001), LN metastasis (*P* < 0.001), and adjuvant therapy (*P* < 0.001) were all prognostic factors for PFS. However, the number of RLN was not a prognostic factor for PFS, either as a categorical variable (*P*=0.183) or as a continuous variable (*P*=0.218). Moreover, in multivariate analysis, the number of RLN was not an independent prognostic factor for PFS, either as a categorical variable (*P*=0.528) or as a continuous variable (*P*=0.523). Analyses for prognostic factors predicting OS showed similar results; the number of RLN was not a prognostic factor either in univariate analysis (*P*_categorical_ = 0.194, *P*_continuous_ = 0.420) or in multivariate analysis (*P*_categorical_ = 0.292, *P*_continuous_ = 0.853).

### 3.5. Subgroup Analyses

**Figure 4:**
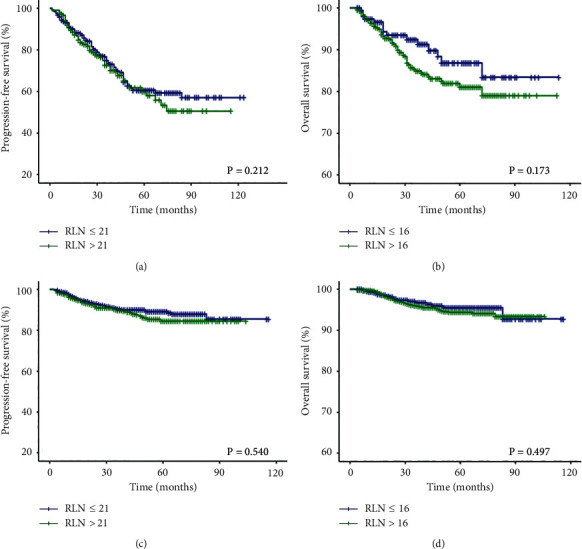
Kaplan-Meier plot of PFS (a) and OS (b) according to the number of RLN in LN positive patients. Kaplan-Meier plot of PFS (c) and OS (d) according to the number of RLN in LN negative patients. PFS, progression-free survival; OS, overall survival; RLN, removed lymph nodes; LN, lymph nodes.

Subgroup analyses were performed for LN positive and LN negative patients. In LN positive cohort (*n* = 668), as shown in Figures 4(a) and 4(b), the number of RLN did not show a statistically significant association with either PFS (*P*=0.212) or OS (*P*=0.173). The univariate and multivariate analyses for PFS and OS showed that the number of RLN is not a prognostic factor, either as a categorical variable (univariate: *P*_PFS_ = 0.542, *P*_OS_ = 0.176; multivariate: *P*_PFS_ = 0.853, *P*_OS_ = 0.419) or as a continuous variable (univariate: *P*_PFS_ = 0.625, *P*_OS_ = 0.763; multivariate: *P*_PFS_ = 0.938, *P*_OS_ = 0.458, Table 3). We then moved from the LN positive cohort to the LN negative cohort and repeated the same analyses. As shown in Figures 4(c) and 4(d), the number of RLN was not significantly correlated with PFS (*P*=0.540) and OS (*P*=0.497). The univariate and multivariate analyses for PFS and OS showed that the number of RLN is not a prognostic factor, either as a categorical variable (univariate: *P*_PFS_ = 0.213, *P*_OS_ = 0.498; multivariate: *P*_PFS_ = 0.199; *P*_OS_ = 0.393) or as a continuous variable (univariate: *P*_PFS_ = 0.353, *P*_OS_ = 0.211; multivariate: *P*_PFS_ = 0.267, *P*_OS_ = 0.295, Table 4).

## 4. Discussion

For cervical cancer, until the new FIGO staging system was established in 2018 [19], LN status did not modify the FIGO stage. However, if LN metastases are present, the 5-year survival rate is to drop from 85% to 50% [20]. In this study, the five-year PFS rate of patients with and without nodal metastasis was 57.1% and 87.4%, respectively, and the five-year OS rate was 82.4% and 94.7%, respectively. Therefore, over time, gynecologic oncologists have been absorbed in pursuing a more thorough lymphadenectomy. The extent of lymph node area has been enlarged, and the completeness of LN dissection has even been the basis for surgical skills. Thus, the potential therapeutic role of lymphadenectomy for patients with early-stage cervical cancer has got scant attention. However, as the treatment of cancer is becoming more and more precise and individualized, surgical oncologists need to calm down and think about whether the patients could benefit from the “aggressive” LN dissections.

There had been some published researches on the lymphadenectomy in cervical cancer, and the effect of the number of RLN on survival in early-stage cervical cancer patients remains questionable. In a Surveillance Epidemiology and End Results (SEER) analysis of 5522 patients with stage IA2-IIA cervical cancer patients who underwent RHPL, Shah et al. concluded that node-negative and early-stage cervical cancer patients attained the better survival from a more extensive lymphadenectomy [14]. In another retrospective study, Kim et al. found the increased number of RLN was associated with better survival in patients treated with surgical treatment compared to those treated with neoadjuvant chemotherapy followed by surgery [21]. However, Pieterse et al. found that there was no relation between the number of RLN and survival for LN negative patients but noted the improved survival in LN positive patients with a higher number of RLN [22]. But, in a study by Shah et al., who also separated patients into LN positive and LN negative groups, a more extensive lymphadenectomy had no effect on survival among patients with positive LN. For patients with negative LN, the higher number of RLN was associated with improved survival [15]. On the contrary, Mao et al. indicated that the number of RLN was not an independent prognostic factor for patients with node-negative early cervical cancer in a study of 359 lymph-node-negative patients with FIGO stage IA-IIB cervical cancer [16].

The same issue led to many inconsistent conclusions noted by other studies [23, 24]. The reason was that not all patients underwent the same extent of dissection. For instance, patients with comorbidities often received less extensive procedures than healthier patients, and the experienced surgeons could perform a more aggressive pelvic node dissection. Besides, except for the surgical approach, the number of RLN may also be affected by some other bias, including the method of LN submission, pathologist's performance, physiologic variation, and inclusion of different pathological types. Analysis of the effects of removing a different number of LN may be biased.

The patients in our study were more homogenous as only CSCC was included, and patients with comorbidities were excluded. Also, we limited our analysis to stage IB-IIA patients and patients with stage IA2 were excluded for the prevalence of positive nodes, or pelvic wall recurrence is much lower in patients with stage IA2 cervical cancer than in patients with stage IB-IIA cervical cancer [25]. In the current study, the number of RLN was not associated with PFS and OS. Besides, Cox regression analysis showed that the number of RLN was not an independent prognostic factor for PFS and OS in all patients, LN positive or LN negative patients, respectively.

In one of our previous studies, we compared the prognostic accuracy of four LN staging systems—the 2018 FIGO stage, number of positive lymph nodes (PLN), metastatic lymph node ratio (LNR), and log odds of positive lymph nodes systems—in patients with node-positive CSCC following radical surgery, and found the PLN system seemed to be the most accurate LN staging method, indicating that the number of PLN rather than the number of RLN is the real factor affecting the prognosis of patients [26]. Therefore, it brings us the thoughts of the necessity for complete lymphadenectomy, which could also bring the related complications. It also brings us the question of how can we accurately assess the LN status preoperatively or intraoperatively? Progress in imaging techniques does provide less invasive methods for identifying lymph node metastases with high accuracy. Some of the new methods reported for diagnosis of the nodal disease include diffusion-weighted imaging [27], FDG PET combined with diagnostic CT [28], contrast-enhanced ultrasound [29], and CT guided 125I seed interstitial implantation [30]. Besides, prediction models for LN metastasis in cervical cancer patients based on clinical and pathological parameters were established [31, 32].

Moreover, recent studies supported the safety and feasibility of sentinel lymph node biopsy for early-stage cervical cancer [33, 34]. Large-scale and prospective studies are to be established to ensure the safety and accuracy of the above approaches to make the treatment of cervical cancer more accurate. More tailored and less invasive approaches should be encouraged to assess the nodal status and to remove suspicious lymph nodes selectively, especially for patients with low risk of lymph node metastasis. On the other hand, for LN positive patients, lymph node micrometastases might continue when fewer nodes were dissected. Patients with higher numbers of LN dissected appeared more likely to have micrometastases removed, which may bring a better survival. However, our findings suggested that even patients with LN positive did not benefit from a higher number of RLN. It should be noted that cervical cancer with other pathological types, instead of CSCC, may have different results. More studies are needed to find out the relationship between the number of RLN and patients' survival among these pathological types. Different therapeutic strategies for these patients may be selected.

We acknowledge that there were several limitations to our study. First, all data were obtained from a single institution, and this may not reflect the status in other centers. Second, as with previous studies, our study is a retrospective study with the possibility of selection bias. However, to date, all studies investigating the number of RLN as a possible prognosticator are retrospective. Accordingly, in the case of cervical cancer, more reliable prospective randomized trials may be required to define whether the number of RLN is associated with survival.

## 5. Conclusions

If a standardized complete lymphadenectomy was performed, the number of lymph nodes removed was not an independent prognostic factor for survival among CSCC patients. Future prospective studies are needed to expand these findings.

## Figures and Tables

**Table 1:** Association between number of RLN and clinical and pathological features.

Variable	Total N	RLN (PFS)		*P* value	RLN (OS)		*P* value
		6–21 N	21–55 N		6–16 N	17–55 N	
Age, years				0.507			0.86
≤50	2013	1078	935		474	1539	
>50	1114	597	517		259	855	
Menopausal status				0.62			0.653
Premenopausal	2102	1119	983		498	1604	
Postmenopausal	1025	556	469		235	790	
FIGO stage				0.914			0.309
IB	1692	908	784		409	1283	
IIA	1435	767	668		324	1111	
Tumor diameter (cm)				<0.001^*∗*^			<0.001^*∗*^
≤4	2166	1206	960		545	1621	
>4	791	377	414		148	643	
Depth of stromal invasion				0.423			0.081
<1/2	1044	570	474		266	778	
≥1/2	2031	1077	954		460	1571	
LVSI				0.789			0.964
Negative	1863	1001	862		438	1425	
Positive	1092	581	511		258	834	
Parametrial invasion				0.198			0.615
Negative	2934	1580	1354		688	2246	
Positive	143	69	74		36	107	
Vaginal margin invasion				0.923			0.139
Negative	2958	1584	1374		686	2272	
Positive	112	61	51		33	79	

RLN, removed lymph nodes; PFS, progression-free survival; OS, overall survival; FIGO, Federation of Gynecology and Obstetrics; LVSI, lymph-vascular space invasion; RLN, removed lymph nodes.

**Table 2:** Cox proportional hazard analysis on prognostic factors related to survival in all the patients.

Variable	PFS							OS						
	Univariate	Multivariate 1			Multivariate 2			Univariate	Multivariate 1			Multivariate 2		
	*P* value	HR	95% CI	*P* value	HR	95% CI	*P* value	*P* value	HR	95% CI	*P* value	HR	95% CI	*P* value
Age, years	0.014	0.897	0.588–1.369	0.614	0.893	0.585–1.364	0.601	0.052						
Menopausal status	0.004	1.223	0.800–1.870	0.353	1.229	0.803–1.880	0.342	0.008	1.255	0.904–1.743	0.174	1.258	0.906–1.747	0.171
FIGO stage	<0.001	1.450	1.123–1.873	0.004	1.450	1.122–1.872	0.004	<0.001	1.321	0.927–1.881	0.123	1.327	0.932–1.889	0.117
Tumor diameter (cm)	<0.001	1.425	1.118–1.816	0.004	1.425	1.118–1.816	0.004	<0.001	1.720	1.237–2.391	0.001	1.747	1.257–2.426	0.001
Depth of stromal invasion	<0.001	2.177	1.498–3.163	<0.001	2.176	1.497–3.162	<0.001	<0.001	4.145	2.133–8.058	<0.001	4.148	2.133–8.065	<0.001
LVSI	<0.001	2.122	1.610–2.798	<0.001	2.121	1.609–2.795	<0.001	<0.001	2.559	1.740–3.765	<0.001	2.556	1.737–3.761	<0.001
Parametrial invasion	<0.001	1.800	1.290–2.510	0.001	1.803	1.294–2.514	0.001	<0.001	1.689	1.069–2.670	0.025	1.697	1.075–2.680	0.023
Vaginal margin invasion	<0.001	2.025	1.366–3.002	<0.001	2.028	1.368–3.007	<0.001	<0.001	2.060	1.210–3.507	0.008	2.040	1.198–3.474	0.009
Lymph node metastasis	<0.001	1.963	1.507–2.555	<0.001	1.962	1.507–2.554	<0.001	<0.001	1.648	1.146–2.370	0.007	1.641	1.142–2.358	0.007
Adjuvant therapy	<0.001	0.522	0.389–0.699	<0.001	0.522	0.390–0.700	<0.001	<0.001	0.512	0.340–0.771	0.001	0.514	0.341–0.773	0.001
No. of RLN	0.183	1.075	0.859–1.344	0.528				0.194	1.236	0.833–1.834	0.292			
No. of RLN (continuous)	0.218				1.005	0.991–1.019	0.523	0.420				1.002	0.981–1.024	0.853

FIGO, Federation of Gynecology and Obstetrics; LVSI, lymph-vascular space invasion; RLN, removed lymph nodes.

**Table 3:** Cox proportional hazard analysis on prognostic factors related to survival in LN positive patients.

Variable	PFS							OS						
	Univariate	Multivariate 1			Multivariate 2			Univariate	Multivariate 1			Multivariate 2		
	*P* value	HR	95% CI	*P* value	HR	95% CI	*P* value	*P* value	HR	95% CI	*P* value	HR	95% CI	*P* value
Age, years	0.713							0.507						
Menopausal status	0.989							0.643						
FIGO stage	0.005	1.260	0.870–1.823	0.221	1.254	0.866–1.815	0.230	0.331						
Tumor diameter (cm)	0.005	1.468	1.052–2.049	0.024	1.464	1.049–2.042	0.025	0.007	1.664	1.057–2.620	0.028	1.712	1.090–2.691	0.020
Depth of stromal invasion	0.002	2.695	1.167–6.222	0.02	2.719	1.176–6.285	0.019	0.046	2.224	0.690–7.164	0.181	2.127	0.659–6.863	0.207
LVSI	0.014	1.610	1.024–2.531	0.039	1.602	1.020–2.516	0.041	0.007	2.458	1.216–4.966	0.012	2.495	1.235–5.042	0.011
Parametrial invasion	<0.001	2.043	1.393–2.998	<0.001	2.033	1.387–2.979	<0.001	0.001	1.721	1.013–2.924	0.045	1.779	1.049–3.016	0.032
Vaginal margin invasion	0.009	1.914	1.104–3.318	0.021	1.914	1.104–3.319	0.021	0.389						
No. of RLN	0.542	0.969	0.699–1.345	0.853				0.176	1.264	0.717–2.229	0.419			
No. of RLN (continuous)	0.625				1.001	0.981–1.021	0.938	0.763				0.989	0.959–1.019	0.458

FIGO, Federation of Gynecology and Obstetrics; LVSI, lymph-vascular space invasion; RLN, removed lymph nodes.

**Table 4:** Cox proportional hazard analysis on prognostic factors related to survival in LN negative patients.

Variable	PFS							OS						
	Univariate	Multivariate 1			Multivariate 2			Univariate	Multivariate 1			Multivariate 2		
	*P* value	HR	95% CI	*P* value	HR	95% CI	*P* value	*P* value	HR	95% CI	*P* value	HR	95% CI	*P* value
Age, years	0.001	0.700	0.391–1.253	0.230	0.689	0.384–1.235	0.211	0.035	0.416	0.188–0.920	0.030	0.411	0.185–0.909	0.028
Menopausal status	<0.001	1.836	1.033–3.262	0.038	1.865	1.048–3.320	0.034	0.001	2.683	1.229–5.854	0.013	2.718	1.247–5.922	0.012
FIGO stage	<0.001	1.702	1.211–2.392	0.002	1.704	1.212–2.395	0.002	<0.001	1.917	1.171–3.137	0.010	1.929	1.178–3.160	0.009
Tumor diameter (cm)	0.074							0.001	1.887	1.183–3.010	0.008	1.873	1.174–2.990	0.008
Depth of stromal invasion	<0.001	1.799	1.175–2.753	0.007	1.799	1.175–2.755	0.007	<0.001	4.276	1.925–9.498	<0.001	4.294	1.932–9.543	<0.001
LVSI	<0.001	2.186	1.571–3.041	<0.001	2.180	1.567–3.033	<0.001	<0.001	2.433	1.535–3.855	<0.001	2.431	1.534–3.853	<0.001
Parametrial invasion	0.046	1.309	0.633–2.707	0.467	1.307	0.632–2.702	0.470	0.003	1.860	0.793–4.359	0.154	1.791	0.766–4.187	0.178
Vaginal margin invasion	<0.001	2.085	1.177–3.694	0.012	2.095	1.182–3.713	0.011	<0.001	3.635	1.801–7.337	<0.001	3.618	1.795–7.294	<0.001
Adjuvant therapy	0.016	0.688	0.466–1.016	0.060	0.687	0.465–1.015	0.059	0.003	0.582	0.333–1.017	0.057	0.581	0.332–1.017	0.057
No. of RLN	0.213	1.220	0.901–1.652	0.199				0.498	1.272	0.733–2.207	0.393			
No. of RLN (continuous)	0.353				1.011	0.991–1.031	0.267	0.211				1.016	0.986–1.047	0.295

FIGO, Federation of Gynecology and Obstetrics; LVSI, lymph-vascular space invasion; RLN, removed lymph nodes.

## Data Availability

The data used to support the findings of this study are included within the article.

## Abbreviations

RLN: Removed lymph nodes
FIGO: International Federation of Gynecology and Obstetrics
CSCC: Cervical squamous cell carcinoma
RHPL: Radical hysterectomy with pelvic lymphadenectomy
PFS: Progression-free survival
OS: Overall survival
LN: Lymph nodes
LVSI: Lymph-vascular space invasion
SCC-Ag: Serum squamous cell carcinoma antigen
CT: Computed tomography
MRI: Magnetic resonance imaging
HR: Hazard ratios
CI: Confidence intervals
